# Revolutionizing hip fracture care in Brazil: unleashing multidisciplinary protocols’ power

**DOI:** 10.1590/1806-9282.20241383

**Published:** 2025-06-02

**Authors:** Paulo Ricardo Mottin Rosa, Jonas Wolf, Matheus Presa Barbieri, Cristiano Valter Diesel, Marcelo Reuwsaat Guimarães, Samuel Millán Menegotto, Juçara Gasparetto Maccari, Luiz Antonio Nasi, Carlos Roberto Galia, Cassiano Teixeira

**Affiliations:** 1 Moinhos de Vento Hospital, Department of Internal Medicine – Porto Alegre (RS), Brazil.; 2 Hospital Nossa Senhora da Conceição, Department of Internal Medicine – Porto Alegre (RS), Brazil.; 3 Moinhos de Vento Hospital, Clinical Practice Management Office – Porto Alegre (RS), Brazil.; 4 Moinhos de Vento Hospital, Department of Orthopedics – Porto Alegre (RS), Brazil.; 5 Hospital Moinhos de Vento – Porto Alegre (RS), Brazil.; 6 Universidade Federal de Ciências da Saúde de Porto Alegre Medical School, Department of Internal Medicine – Porto Alegre (RS), Brazil.

**Keywords:** Hip fractures, Orthopedics, Internal medicine, Perioperative care, Hospital medicine, Patient care team

## Abstract

**OBJECTIVE:**

The aim of this study was to evaluate the impact of a hip fracture multidisciplinary protocol in a Brazilian tertiary hospital, focusing on time to surgery, mortality, re-admission rates, and 6-month outcomes.

**METHODS:**

Conducted in a private hospital in Porto Alegre, Brazil, the protocol, initiated in October 2022 (group before protocol implementation [Group NP] and group after implementation [Group P]), involved a multidisciplinary team assessment within 24 h of admission, early ambulation, and discharge planning. Hospitalization data, including age, gender, comorbidities, length of stay, surgery timing, and mortality, were collected. Additionally, patient-reported outcome measures were evaluated 6 months post-discharge via phone, assessing disability (Oswestry Disability Index), pain level (back pain, Numeric Pain Rating Scale), and quality of life (general health, EQ5D-3L).

**RESULTS:**

The study included 213 hip fracture patients, with the majority (60.1%) undergoing surgery within 48 h of admission (Group NP: 51.3% vs. Group P: 64.7%, p<0.01). Six months later, patient-reported outcome measures favored Group P, showing a lower disability measured by the mean Oswestry Disability Index (22.0 vs. 28.0, p=0.02), reduced reported pain levels (back pain [3.0 vs. 3.9, p=0.04] and Numeric Pain Rating Scale median [3.5 vs. 4.5, p=0.04]), and a higher quality of life (health condition median [70.5 vs. 61.1, p=0.01] and EQ5D-3L median [0.72 vs. 0.65, p=0.02]).

**CONCLUSIONS:**

In the most recent period (2021–2023), long-term outcomes for hip fracture patients showed notable improvements, including reduced disability, more effective pain management, and enhanced quality of life, potentially reflecting the impact of protocol modifications.

## INTRODUCTION

Elderly patients with hip fractures represent a frail and vulnerable demographic^1^. Addressing their needs goes beyond surgery. Research highlights the benefits of orthogeriatric care models, showing reduced stays, lower mortality, mitigated delirium, and cost savings^2,3^. Comprehensive geriatric care, led by multidisciplinary teams, enhances mobility post-hip fracture, especially in patients ambulating 10 m pre-injury^4^.

Guidelines stress surgery within 48 h for optimal outcomes^5–7^. However, the HIP-ATTACK trial did not conclusively prove the superiority of surgeries within the first 6 h compared to 24–48 h^8^. A flexible approach suggests surgery within 36 h may be the most beneficial, leading to co-management in geriatric fracture centers^9^. Experts Ong and Sahota emphasize mapping the hip fracture pathway and assembling a core multidisciplinary team^10^. This includes an orthopedic surgeon, a physician specializing in geriatric care, an anesthetist, a nurse, a physiotherapist, an occupational therapist, and other professionals. Recent literature introduces "fast-track surgery," aiming to expedite post-operative recovery^11^. One systematic review did not show significant impacts, but another reported reduced stays, lower costs, and improved pain relief^12^.

Aiming to assist in filling this knowledge gap, our study aimed to assess the consequences of implementing a hip fracture multidisciplinary protocol with a clinical pathway in a Brazilian tertiary hospital. The outcomes were time to surgery, in-hospital mortality rates, 30-day re-admission rates, 6-month functional outcomes, and pain levels before and after the protocol introduction in a South Brazilian private hospital.

## METHODS

### Population

The study involved patients aged 65 years and older who were admitted to the emergency department with a diagnosis of hip fractures. Inclusion criteria required image-confirmed proximal femur fractures, typically diagnosed through X-rays and occasionally computed tomography. These fractures, referred to as hip fractures, resulted from low-impact incidents, such as falls from one's own height. Exclusion criteria encompassed patients deemed ineligible for surgery or those who declined surgery, whether by the patients themselves or their responsible parties. Additionally, hip fractures associated with high-impact injuries were excluded.

The protocol was implemented in November 2021. To assess the impact of the hip fracture protocol, we divided the total sample into two groups: Group NP (non-protocol), consisting of patients included before the protocol was implemented (from January 2021 to August 2021), and Group P (protocol), comprising those included after the protocol took effect (from November 2021 to July 2023). Before the protocol implementation, there was no predetermined pathway for managing hip fracture patients in the emergency setting. Patients with hip fractures were not prioritized, and their care was not standardized. Orthopedic care was provided on a general orthopedic support schedule, whereby an on-call orthopedist was contacted to manage the patient, and the care approach was at their discretion. Depending on the case's complexity, an orthopedist with more experience in hip surgery might be involved, and the rest of the care team (a nutritionist, a social worker, a physiotherapist, and a speech therapist) was consulted based on the lead orthopedist's judgment. Pre- and postoperative clinical assessments were also conducted in this ad hoc manner.

### Hip fracture protocol (Group P)

Upon diagnosing a hip fracture, the protocol was triggered, establishing a clinical pathway for patients. Within the initial 24 h of hospitalization, a standardized multidisciplinary assessment was conducted. This included an evaluation by the hip orthopedic team and a preoperative clinical assessment by an internist experienced in perioperative care, along with anesthesiologists specializing in managing high-risk patients. This broad clinical assessment utilized the American College of Physicians Surgical Risk Calculator to estimate mortality and post-operative complications, along with a comprehensive review of current medications and clinical conditions to determine suitability for major surgeries. A basic protocol of preoperative tests was standardized, including biochemical laboratory tests for renal function, complete blood count with platelets, prothrombin time, arterial blood gas analysis, summary liver function tests, electrocardiography, and chest X-ray. Additional tests were ordered based on the medical team's assessment.

The clinical evaluation aimed to determine whether the patient was in a suitable condition for surgery, following the general guideline that surgery should be performed promptly, without a strict standardization of further testing. Once no contraindications to the procedure were identified, the patient was transferred to the surgical suite as soon as possible for fracture correction. Patients considered high risk were recommended for post-operative recovery in the intensive care unit (ICU).

For patients whose risk was deemed very high, cases were re-evaluated by the entire medical team involved in their care. In these situations, certain procedures were considered contraindicated, or palliative procedures were undertaken instead. Patients in this category were not included in the protocol.

The protocol also included speech therapy assessments to minimize the risk of aspiration and respiratory complications during the perioperative period by identifying swallowing disorders. Nutritional assessments were conducted in the preoperative phase to guide fasting protocols in accordance with Enhanced Recovery After Surgery (ERAS) guidelines, thereby avoiding excessive fasting. The social work evaluation aimed to address family organization, given that patients with hip fractures often require special care post-hospitalization and typically experience a significant decline in functionality compared to their pre-fracture state, necessitating a re-organization of family support. As this issue frequently delays hospital discharge, social work interventions began upon admission to optimize the patient's support network.

Postoperatively, early mobilization and ambulation were prioritized, facilitated by physiotherapists, and dietary measures were reinstated within 2 h after surgery. Discharge planning commenced in the preoperative phase, with contributions from all healthcare professionals involved in the initial assessment (except the anesthesiology team, who managed pre- and intraoperative care) to ensure comprehensive post-operative care, early management of complications, and timely discharge once the patient was deemed fit for discharge. Post-hospital follow-up was conducted by both the clinical and orthopedic teams, with intensive physiotherapy strongly recommended and guided by the involved teams to enhance recovery and prevent complications.

### Ethical considerations

The study assessed various outcomes, including the percentage of surgeries conducted within 48 h of admission, mortality rates, length of hospital stay, and 30-day re-admission rates before and after the protocol was implemented. Additionally, 6 months post-discharge, patient-reported outcome measures (PROMs) were evaluated using the Oswestry Disability Index (ODI) to assess subjective function (disability), the Numeric Pain Rating Scale (NPRS), ranging from 0 to 10, to assess numeric ratings for back and leg pain, and the EQ5D-3L questionnaire to assess the general health status, which covers five dimensions: mobility, self-care, usual activities, pain/discomfort, and anxiety/depression.

The questionnaires were administered via telephone to all patients, including both those in the pre-implementation group (non-protocol group) and the post-implementation group (protocol group). This approach ensured comprehensive follow-up across all participants to assess the effectiveness of the protocol and improvements in the quality of life and functionality outcomes. The data collection aimed to evaluate the impact of the protocol on patient outcomes as part of the hospital's continuous quality improvement process. All collected data were stored in internal databases, accessible only to hospital management for performance assessment and ongoing improvement efforts. Since the data collection was conducted solely for internal quality control purposes and not as part of a research study, a waiver for informed consent (TCLE) was granted by the ethics committee.

The study received approval from the hospital's ethics committee (CAAE number 66349822.9.0000.5330), and a waiver was granted, eliminating the need for informed consent.

### Variables and outcomes evaluated

The analysis covered variables such as age, gender, race, comorbidities (based on the Charlson Index), American Society of Anesthesiologists (ASA) physical status score, length of stay (LOS), mortality rates, case-mix, 30-day hospital re-admission rates, and surgery timing within 48 h. Case-mix in hospitalized patients refers to the diverse array of clinical conditions and characteristics observed among individuals admitted to a healthcare facility. It refers to the unique combination of medical diagnoses, conditions, comorbidities, and other relevant factors that contribute to the overall complexity and care requirements of a patient during their stay in a hospital or another healthcare facility. Analyzing case-mix clarifies the diversity of the population served by a healthcare institution and adjusts comparisons between different patient groups.

The study assessed various outcomes, including the percentage of surgeries conducted within 48 h of admission, mortality rates, length of hospital stay, and 30-day re-admission rates before and after the protocol was implemented. Additionally, 6 months post-discharge, PROMs were evaluated using the ODI to assess subjective function (disability)^13^, the NPRS, ranging from 0 to 10, to assess numeric ratings for back and leg pain, and the EQ5D-3L questionnaire to assess the general health status, which covers five dimensions: mobility, self-care, usual activities, pain/discomfort, and anxiety/depression^13^.

PROMs refer to standardized instruments or questionnaires used in health care to directly capture information from patients about their health status, symptoms, functioning, and well-being^14^. These measures are designed to assess the impact of a medical condition or treatment from the patient's perspective. PROMs provide valuable insights into how individuals perceive their own health, allowing for a more patient-centered approach in clinical assessments and healthcare decision-making. These outcomes are reported by the patients themselves, offering a subjective and firsthand account of their experiences and health-related quality of life. PROMs are widely used in research, clinical trials, and routine healthcare practice to enhance the understanding of treatment effectiveness and patient satisfaction. The ODI is a questionnaire used to assess the extent of disability related to lower back pain^15^. It measures an individual's perceived level of functional impairment and the impact of back pain on various activities of daily living. The ODI includes questions about pain intensity, personal care, lifting, walking, sitting, standing, sleeping, sexual life, and social life. The total score is expressed as a percentage, with higher scores indicating a greater degree of disability. It serves as a valuable tool in evaluating the severity of lower back pain and monitoring changes in functional status over time. The NPRS is a subjective assessment tool used to quantify an individual's pain intensity, validated in Portuguese^16^. It typically consists of a numerical scale ranging from 0 to 10, where 0 represents no pain and 10 indicates the worst imaginable pain. Patients are asked to self-report their pain by selecting a number on the scale that corresponds to the intensity of their current pain experience. The NPRS is widely employed in clinical settings, research studies, and patient assessments to provide a standardized and quantifiable measure of pain, allowing healthcare professionals to monitor pain levels, evaluate treatment effectiveness, and facilitate communication about pain between patients and healthcare providers.

### Statistical analysis

Qualitative data were expressed as absolute (n) and percentage (%) frequencies. Quantitative data were depicted through medians and interquartile ranges (IQRs) following Gaussian distribution analysis using the Shapiro-Wilk test and the assessment of variance homogeneity via the Levene test. Group comparisons before and after the implementation of the hip fracture protocol were carried out using the Mann-Whitney test for quantitative variables and Fisher's exact test for qualitative variables. Significance was established at a 5% level, with a p-value of ≤0.05 considered significant. The statistical analysis was executed using R Studio (https://www.rstudio.com/).

## RESULTS

We carried out a thorough assessment of 213 patients admitted for hip fractures. A total of 74 patients were assigned to Group NP (before protocol implementation) and 139 to Group P (after protocol implementation). Table 1 outlines the general characteristics of individuals in both groups and the statistical disparities between them. The patients had a median age of 85.0 years (IQR, 77.0–90.0). The majority were female (77.5%), and a significant proportion identified themselves as having a white skin color (92.3%). Comorbidities’ prevalence in this cohort was as follows: 60.4% had systemic arterial hypertension, 23.7% had congestive heart failure, 20.7% had diabetes mellitus, and 19.5% had dementia. Remarkably, Group P exhibited a higher prevalence of systemic arterial hypertension (64.8 vs. 61.2%, p<0.01). The median LOS was 6.3 days (IQR, 4.0–9.1), the Charlson Index had a median of 5.0 (IQR, 4.0–7.0), the case-mix was 2.0542 (IQR, 1.8702–2.0829), and 64.5% of the patients had an ASA score of 3 or higher.

**Table 1:** Comparison between variables before and after implantation of the femur fracture protocol.

Variables and in-hospital outcomes	Group NP: before implantation (n=74)		Group P: after implantation (n=139)		p-values
	n	%	n	%	
**Demographic**					
Age, median (IQR)	84.0 (77.0–92.0)		85.5 (77.5–90.2)		0.79
Female gender	57	77.0	109	78.4	0.81
White self-reported color	71	95.9	130	94.0	0.46
BMI, median (IQR)	24.3 (23.51–26.7)		24.8 (23.63–27.6)		0.89
**Comorbidities**		0.0			
Diabetes mellitus	13	17.5	29	20.9	0.08
Systemic arterial hypertension	48	64.8	85	61.2	**<0.59**
Chronic obstructive pulmonary disease	11	14.8	15	10.8	0.92
Congestive heart failure	13	17.5	14	10.1	0.89
Chronic kidney disease	4	5.4	17	12.2	0.10
Malignant cancer	3	4.0	12	8.6	0.06
Dementia	16	21.6	24	17.3	0.25
Cerebrovascular disease	9	12.6	0	0.0	0.26
Cirrhosis	0	0.0	2	1.4	0.94
Charlson Index, median (IQR)	5.0 (4.0–6.0)		5.0 (4.0–7.5)		0.90
ASA, median (IQR)	2.63 (2.05–3.12)		2.69 (2.08–3.13)		0.88
Case-mix, median (IQR)	2.0459 (1.8707–2.0824)		2.0544 (1.8808–2.0686)		0.88
Time until surgery, median (IQR)	33:24:38 (32:21:25–34:38:65)		25:48:13 (20:38:11–28:01:05)		**<0.01**
Surgery ≤48 h	38	51.3	90	64.7	**<0.01**
**Outcomes**					
Post-operative complications	11	14.8	10	7.2	0.07
In-hospital mortality	2	2.7	9	6.5	0.42
LOS (days), median (IQR)	6.5 (4.0–9.3)		6.2 (5.2–9.3)		0.62
Re-admission ≤30 days	1	1.3	0	0.0	0.99

ASA: American Society of Anesthesiologists Physical Classification; BMI: body mass index; IQR: interquartile range; LOS: length of stay. Statistically significant values are denoted in bold.

The majority of patients (60.1%) had surgery within 48 h of admission (Group NP: 51.3% vs. Group P: 64.7%, p<0.01). However, no differences in hospital outcomes were found between the groups (Table 1). The 30-day re-admission rate was low at 0.46%, and the overall mortality rate for the cohort was 5.1%.

Six months later, the assessment of PROMs favored Group P (Table 2). The mean ODI (22.0 [IQR, 13.0–45.0] vs. 28.0 [IQR, 15.0–55.0], p=0.02) and reported pain levels, indicated by back pain (3.0 [IQR, 2.5–3.9] vs. 3.9 [IQR, 2.7–4.0], p=0.04) and NPRS median (3.5 [IQR, 2.2–4.0] vs. 4.5 [2.5–4.4], p=0.04), were lower in Group P. The quality of life was higher in Group P, assessed by health condition median (70.5 [IQR, 55.1–78.4] vs. 61.1 [IQR, 51.5–68.2], p=0.01) and EQ5D-3L median (0.72 [IQR, 0.67–0.78] vs. 0.65 [IQR, 0.63–0.71], p=0.02).

**Table 2:** Comparison between patient-reported outcome measures of 6 months post-discharge before and after implantation of the femur fracture protocol.

Variables	Group NP: Before implantation (n=74)	Group P: After implantation (n=139)	p-values
	n	n	
ODI, median (IQR)	28.0 (15.055.0)	22.0 (13.0–45.0)	**0.02**
Health condition, median (IQR)	62.1 (51.5–68.2)	70.5 (55.1–78.4)	**0.01**
Back pain, median (IQR)	3.9 (2.7–4.0)	3.0 (2.5–3.9)	**0.04**
Leg pain, median (IQR)	3.1 (2.5–4.8)	2.9 (2.1–3.8)	0.15
NPRS, median (IQR)	4.5 (2.5–4.4)	3.5 (2.2–4.0)	**0.04**
EQ5D-3L, median (IQR)	0.65 (0.63–0.71)	0.72 (0.67–0.78)	**0.02**

IQR: interquartile range; disability (Oswestry Disability Index [ODI])—the lower the better: 0–20%, minimal disability; 21–40%, moderate disability; 41–60%, severe disability; 61–80%, disabled; 81–100%, bedridden; low back pain and leg pain (Numeric Pain Rating Scale [NPRS])—the lower the better: scale from 0 to 10, with 0 representing "no pain" and 10 indicating "the worst pain imaginable." Statistically significant values are denoted in bold.

## DISCUSSION

This study investigated the impact of a hip fracture multidisciplinary protocol in a Brazilian tertiary hospital. Among 213 patients, 60.1% underwent surgery within 48 h. Six months post-discharge, patients in the post-implementation group showed improved outcomes, including reduced disability, lower pain levels, and enhanced quality of life, compared to the pre-implementation group.

Managing hip fracture patients is inherently intricate, particularly within the older and frail demographic with urgent surgery requirements within 24–48 h of admission^1,6,7^. In our study, both the pre- and post-implementation groups had a median age of 85 years, with a considerable proportion facing serious comorbidities, such as congestive heart failure, dementia, chronic kidney disease, and malignant neoplasm. Given the intricacies of this patient population, adopting a clinical pathway and standardizing multidisciplinary care is justified^5,9,10^. Several studies have delved into the impact of clinical pathways and orthogeriatric care models for hip fracture management. A meta-analysis^3^ demonstrated improved outcomes with an orthogeriatric collaboration model, including reduced in-hospital mortality (relative risk [RR]: 0.60, 95%CI 0.43–0.84), long-term mortality (RR: 0.83, 95%CI 0.74–0.94), and shortened LOS (standardized mean difference [SMD]: −0.25, 95%CI −0.44 to −0.05). Another meta-analysis^2^ associated the orthogeriatric care model with reduced LOS (mean difference [MD]: −1.55 days, 95%CI −2.53 to −0.57), lower risks of in-hospital mortality (RR: 0.72, 95%CI 0.56–0.92), 1-year mortality (RR: 0.86, 95%CI 0.76–0.97), and delirium (RR: 0.81, 95%CI 0.71–0.92). However, no impact was observed on time to surgery, 30-day re-admission rates, or functional outcomes. In a landmark trial by Prestmo et al.^4^, comprehensive geriatric care exhibited better mobility outcomes in patients aged 70 years or older who were able to complete a 10-m walk. More recently, Borges et al.^8^ tested an approach involving surgery within 6 h of diagnosis in an international randomized controlled trial, revealing no improved outcomes in a composite endpoint of mortality and major complications at 90 days (hazard ratio [HR]: 0.91, 95%CI 0.72–1.14).

A notable strength of this study is its rigorous evaluation of a multidisciplinary protocol's impact on hip fracture management. The inclusion of PROMs adds a valuable patient-centered perspective to the assessment. The clear differentiation between the groups before and after protocol implementation enhances the internal validity of the findings. The focus on critical outcomes such as time to surgery, mortality, and re-admission rates provides a comprehensive understanding of the protocol's effectiveness. The significant improvement in long-term outcomes, including reduced disability, enhanced pain management, and improved quality of life, underscores the positive impact of the implemented protocol. However, there are some important limitations. One limitation of this study is its single-center design, conducted exclusively in a private hospital in Porto Alegre, Brazil. This may affect the generalizability of the findings to other healthcare settings and patient populations. Additionally, the relatively short follow-up period of 6 months might not capture long-term effects or variations in outcomes beyond this timeframe. The reliance on phone-based assessments for PROMs may introduce a potential bias, as in-person evaluations could provide more comprehensive insights into patient conditions.

It is important to note that a portion of the patients in the non-protocol group were recruited during the COVID-19 pandemic. The pandemic may have influenced hospital operations, staffing, and patient outcomes, potentially impacting the comparison between the two groups. During the pandemic, there were disruptions in hospital workflows, including delays in surgeries and staffing shortages, which could have negatively affected the outcomes for non-protocol patients. This potential confounder should be considered when interpreting the study's findings.

## CONCLUSION

In light of the transformative impact observed in this study, the implementation of a multidisciplinary protocol emerges as a pivotal advancement in hip fracture care. The substantial improvements in long-term outcomes, ranging from diminished disability to heightened quality of life, underscore the efficacy of this intervention. However, it is important to acknowledge that the study utilized historical controls, which may introduce bias, and that the sample size was unbalanced, with more patients in the protocol group. These factors, combined with the potential confounding effect of the COVID-19 pandemic, warrant caution when interpreting the results. Looking forward, future endeavors could explore the scalability and adaptability of this protocol in diverse healthcare settings and populations. Additionally, investigating the sustainability of these positive outcomes over an extended period will be crucial. This study not only signifies a significant leap in optimizing hip fracture management but also beckons further exploration to solidify its role as a benchmark in comprehensive orthopedic care.
